# Supplementary material for: HEB is required for the specification of fetal IL-17-producing γδ T cells
Source: Nat Commun. 2017 Dec 8;8:2004. doi: 10.1038/s41467-017-02225-5 (PMC5722817; doi:10.1038/s41467-017-02225-5)
Supplement: Supplementary file 1 — Supplementary Information [file 41467_2017_2225_MOESM1_ESM.pdf]

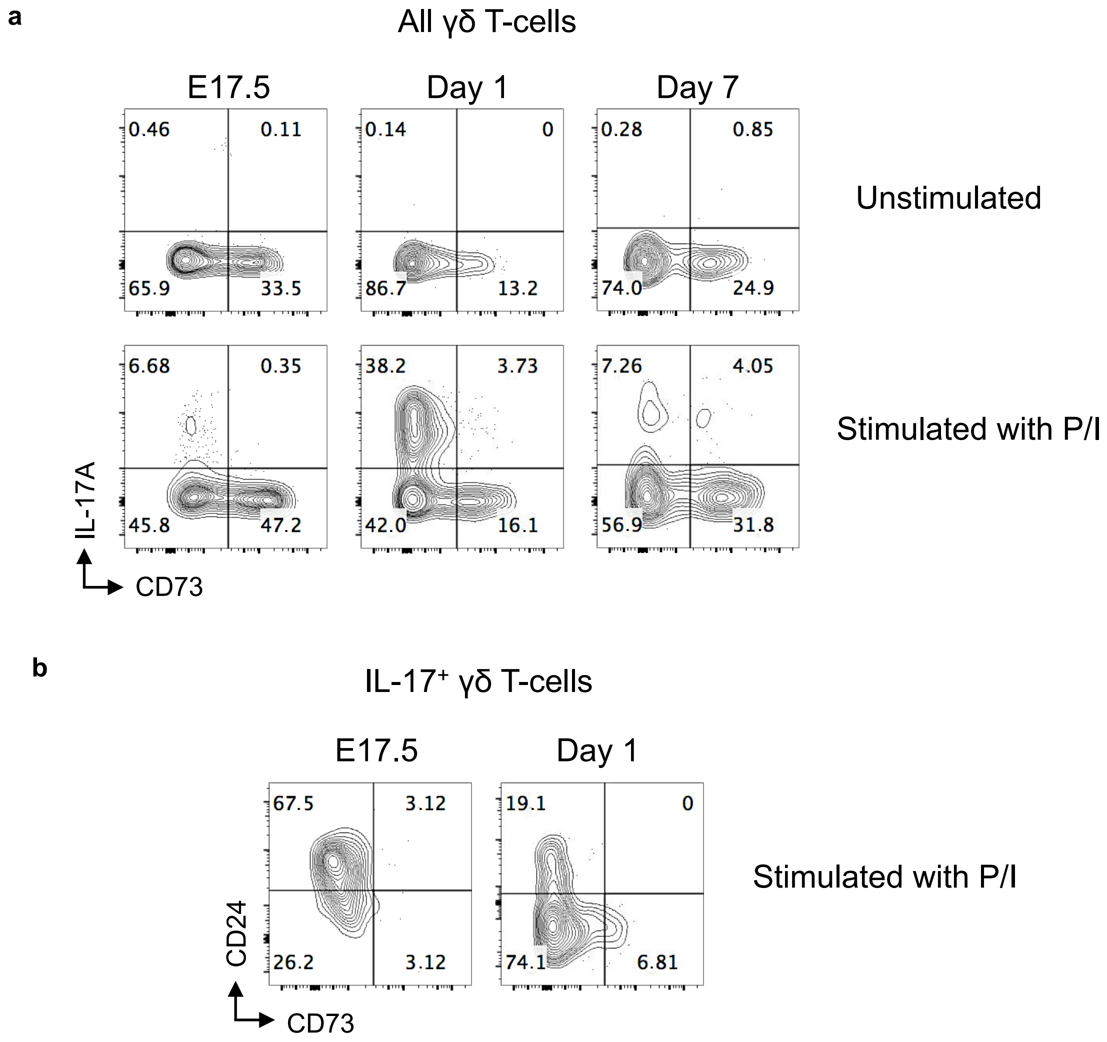

Supplementary Figure 1. Identification of a novel IL-17-producing γδ T-cell subset in WT fetal and neonatal thymus. a. Intracellular staining for IL-17A and surface staining for CD73 in all γδ T cells from E17.5, Day 1 and Day 7 ex vivo WT thymus after 5 hours of stimulation with PMA and Ionomycin (P/I). **b**. Developmental profiles of IL-17A^{+} γδ T-cells. Numbers in the FACS plots indicate frequency within each quadrant. Data are representative of at least 3 mice. All plots are gated on CD3^{+}TCRγδ^{+} cells.

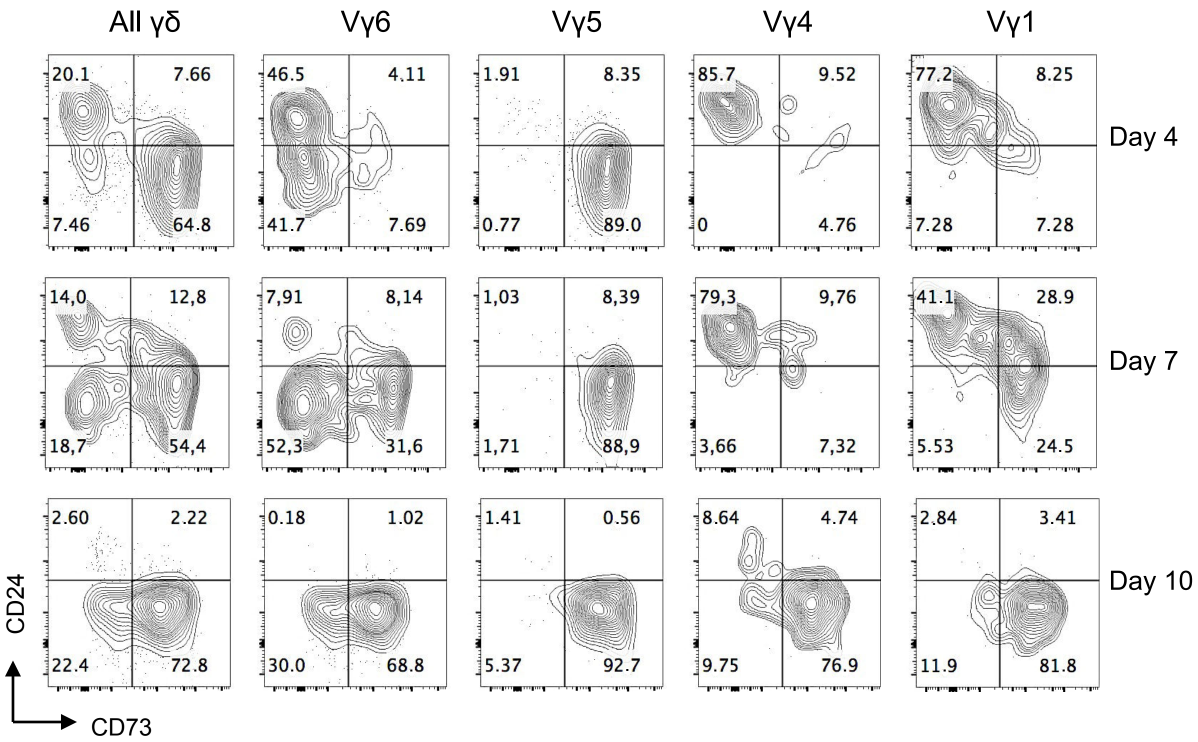

Supplementary Figure 2. Developmental snapshot of Vγ subsets during WT FTOC. Expression of CD24 and CD73 in total γδ T-cells ($CD3^{+}TCR\gamma\delta^{+}$), Vγ6 ($V\gamma5^{-}V\gamma4^{-}V\gamma1^{-}$) γδ T cells, Vγ5 ($V\gamma5^{+}$) γδ T cells, Vγ4 ($V\gamma4^{+}$) γδ T cells and Vγ1 ($V\gamma1^{+}$) γδ T cells in E14.5 FTOC day 4, 7 and 10, as determined by flow cytometry. Numbers in the FACS plots indicate frequency in each quadrant. All plots are gated on $CD3^{+}TCR\gamma\delta^{+}$ cells.

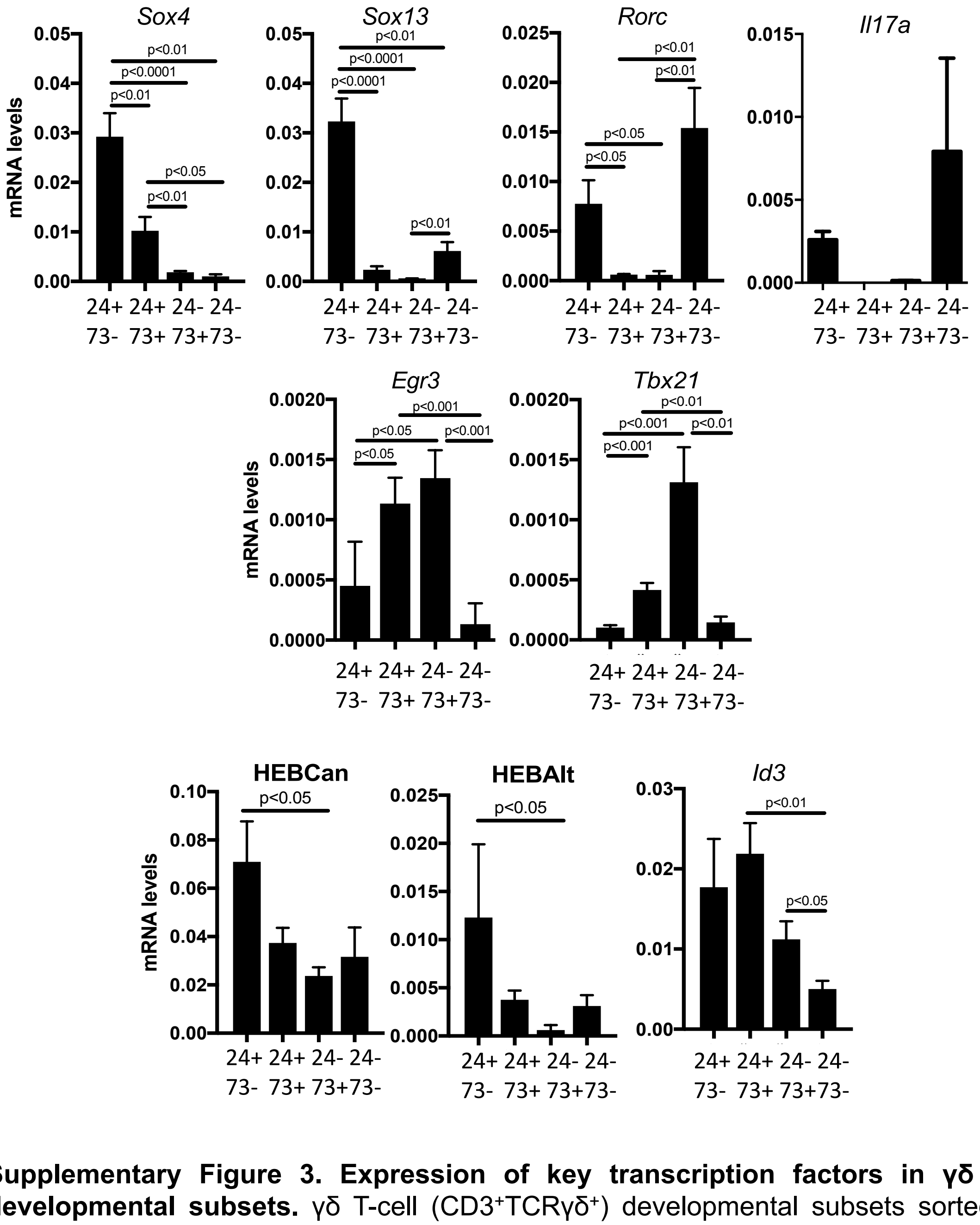

Supplementary Figure 3. Expression of key transcription factors in γδ T-cell developmental subsets. γδ T-cell ($CD3^{+}TCR\gamma\delta^{+}$) developmental subsets sorted from WT E14.5 FTOC day 7 and analyzed for the expression of genes involved in γδT17 cell or γδT1 cell development, and HEB and Id3 factors. All mRNA values were normalized to β-actin. Data are representative of at least 3 independent experiments with 3 mice per group. Unless indicated, differences between samples are not statistically significant. Values indicate mean, error bars denote mean+/-SEM. *p* values are given for all significant differences as determined by two tailed Student t test.

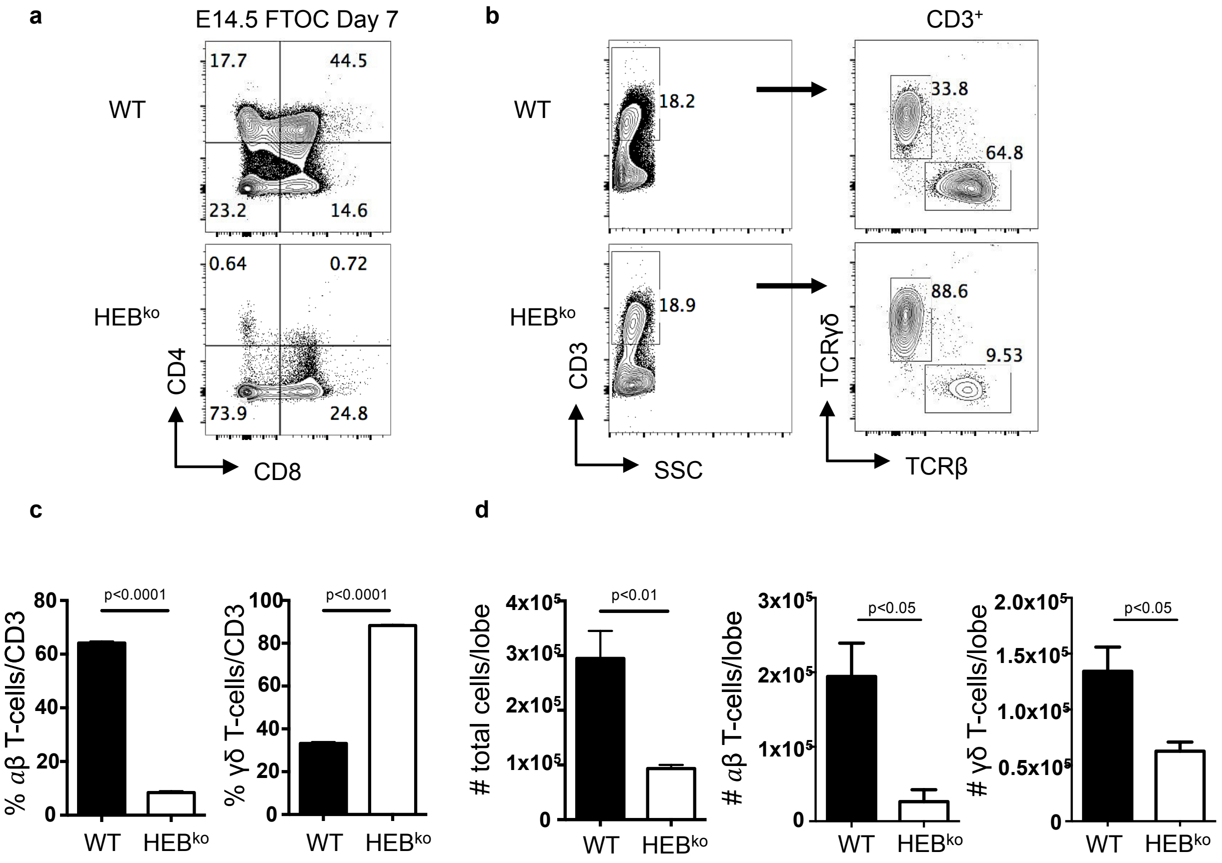

Supplementary Figure 4. γδ T-cells develop in HEB KO fetal thymus. Single cell suspensions were prepared from WT and HEB^{ko} E14.5 FTOC day 7 and subjected to flow cytometry. **a.** Representative flow cytometry plot of CD4 and CD8 expression. **b**. Representative flow cytometry plot showing frequencies of CD3^{+} cells among all cells, and of γδ T-cells (TCRγδ^{+}) cells and αβ T-cells (TCRβ^{+}) within the CD3^{+} gate. **c.** Quantification of frequencies of γδ T-cells and αβ T-cells among total T cells gated. **d.** Absolute numbers of total thymocytes, αβ T-cells, and γδ T-cells, calculated from trypan blue counting and flow cytometry frequencies. Data are representative of at least 3 independent experiments with at least 3 mice per group. Numbers in FACS plots indicate the frequency within each quadrant or gate. Values indicate mean, error bars denote mean+/-SEM. *p* values are given for all significant differences as determined by two tailed Student t test.

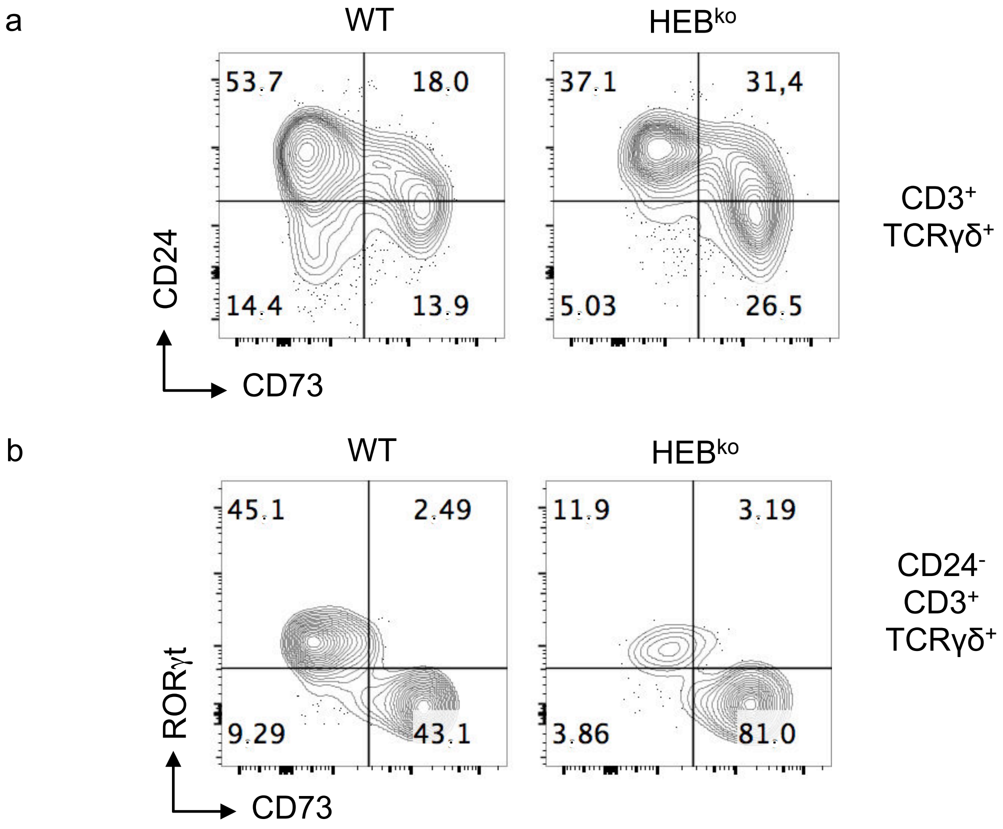

Supplementary Figure 5. Flow cytometric analysis of E17.5 ex vivo WT and HEB^{ko} thymocytes. a. Expression of CD24 and CD73 within all γδ T-cells from E17.5 ex vivo WT and HEB^{ko} thymus. **b.** Expression of RORγt in the mature CD73^{+} and CD73^{-} populations, as assessed by intracellular staining. All plots are gated on CD3^{+}TCRγδ^{+} cells; plots are also gated on CD24^{-} cells in (b). Numbers in FACS plots indicate frequency within each quadrant.

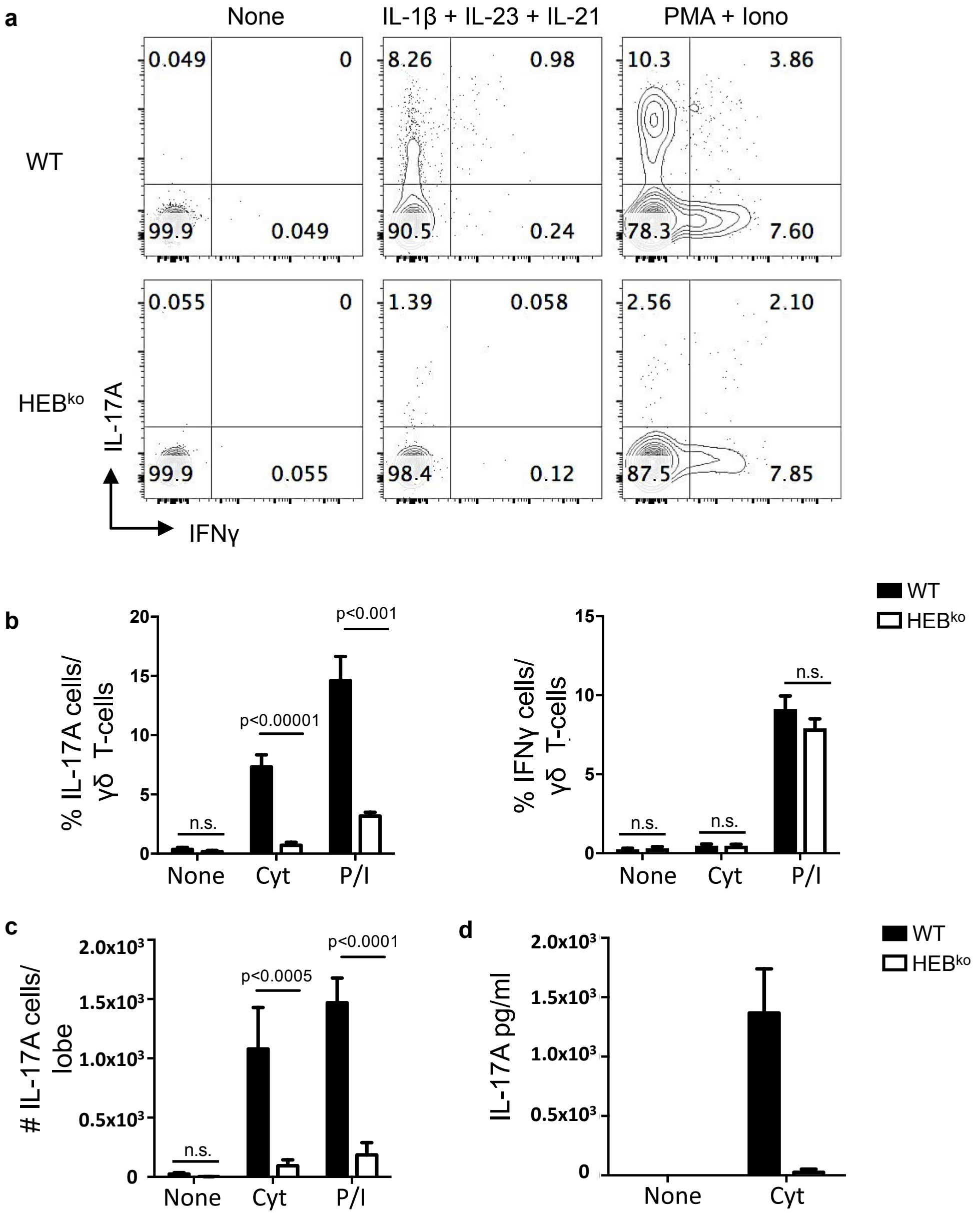

Supplementary Figure 6. HEB^{ko} fetal thymic γδ T-cells are defective in IL-17A production. a. Representative FACS plots of intracellular staining for IL-17A in all γδ T-cells from WT and HEB^{ko} E14.5 FTOC day 7 after 5 hours of stimulation with cytokines (Cyt: IL-1β, IL-23, IL-21 and IL-7) or PMA and Ionomycin (P/I: PMA plus Ionomycin and IL-7). Numbers in FACS plots indicate frequency within each quadrant. **b.** Frequencies of IL-17A^{+} γδ T-cells and IFNγ^{+} γδ T-cells among total γδ T-cells, gated as in (**a**). **c.** Absolute numbers of IL-17A^{+} γδ T-cells/thymic lobe. Graphs in (a, b, c) were calculated to include both single (IL-17 or IFNγ) producers and double (IL-17 and IFNγ) producers. **d.** Concentration of IL-17A produced by WT or HEB^{ko} γδ T-cells after 72 hours of stimulation with IL-1β, IL-23, IL-21 and IL-7, as determined by ELISA. Data are representative of at least three independent experiments with three mice per group in each. Values indicate mean, error bars denote mean+/-SEM. *p* values are given for all significant differences as determined by two tailed Student t test. n.s.=not significant.

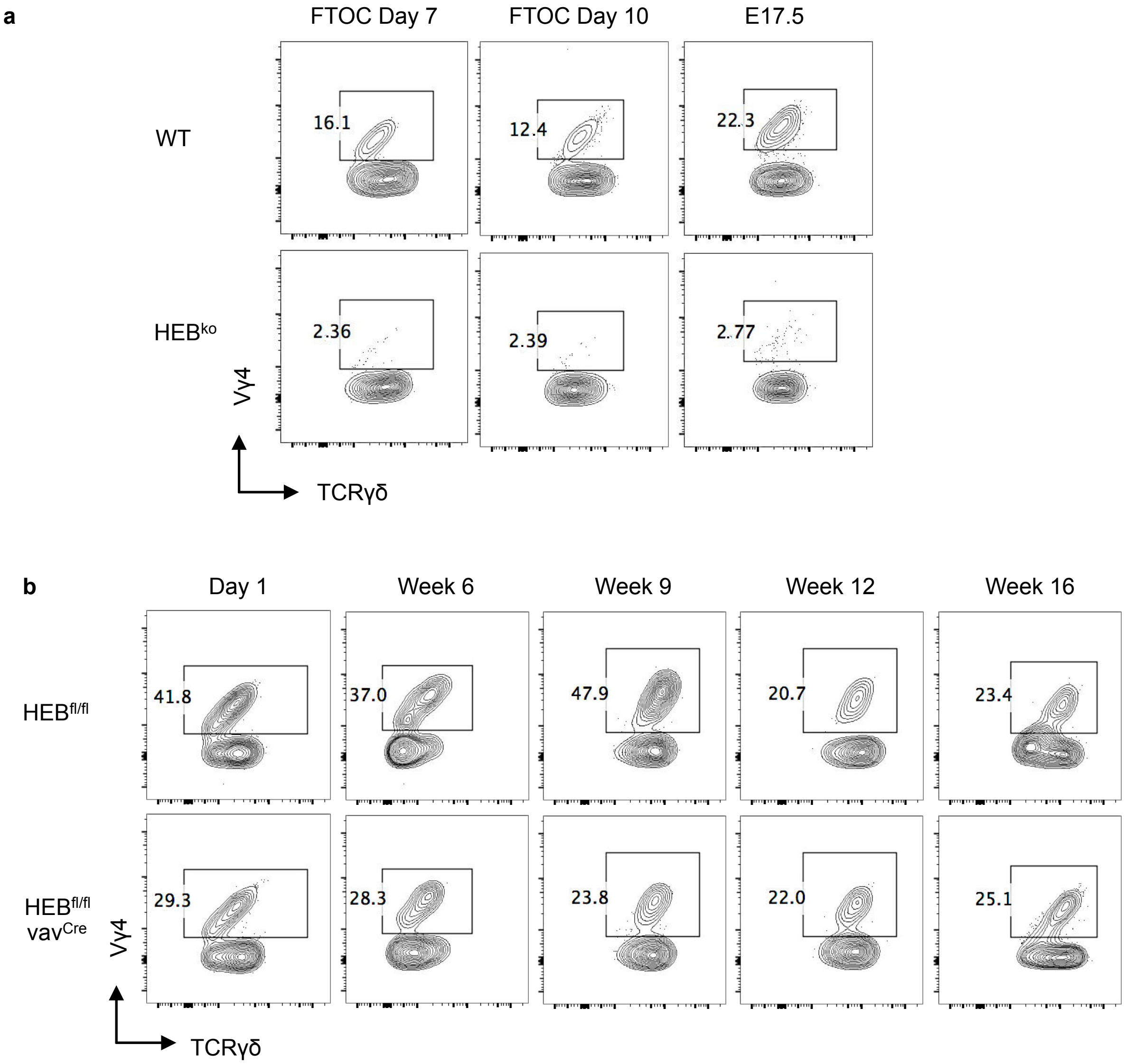

Supplementary Figure 7. Vγ4^{+} cells are present in postnatal thymus of HEB^{ko} mice. a. Flow cytometry analysis of Vγ4 expression in fetal thymus within CD3^{+}TCRγδ^{+} cells from WT and HEB^{ko} E14.5 FTOC day 7 and 10, and E17.5 ex vivo thymus. **b.** Vγ4^{+} cells among CD3^{+}TCRγδ^{+} cells from the ex vivo thymus of HEB$^{fl/fl}$ and HEB$^{fl/fl}$ vav^{Cre} mice at the indicated ages. Numbers in FACS plots indicate frequency in each gate. Data are representative of at least three mice per group.

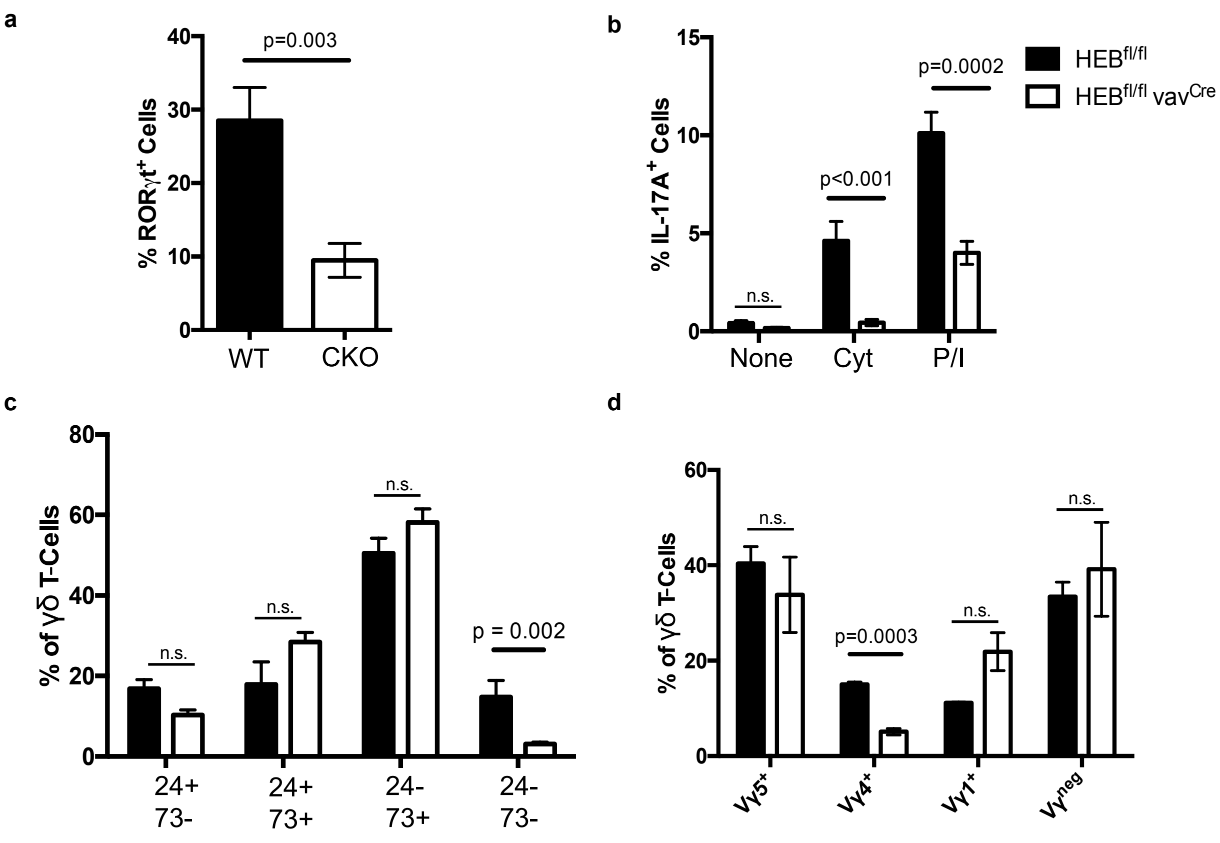

Supplementary Figure 8. γδT17 development in E14.5 FTOC day 7 of HEB$^{fl/fl}$ vav^{Cre} mice. a. Frequency of RORγt^{+} cells in total γδ T-cells (CD3^{+}TCRγδ^{+}) as determined by intracellular flow cytometry. **b.** Frequency of IL-17A^{+} cells in total γδ T-cells stimulated for 5 hours with cytokines (IL-1β, IL-23, IL-21 and IL-7) or P/I (PMA plus Ionomycin and IL-7), as determined by intracellular flow cytometry. **c.** Frequency of γδ T-cells in CD24/ CD73 subsets. **d.** Frequency of Vγ subsets in total γδ T-cells as determined by flow cytometric analysis of surface Vγ expression. All analyses were conducted on thymocytes from FTOC day 7 from HEB$^{fl/fl}$ and HEB$^{fl/fl}$ vav^{Cre} E14.5 embryos. Data are representative of at least three independent experiments with at least three mice per group. Values indicate mean, error bars denote mean+/-S.E.M. *p* values are given for all significant differences as determined by two tailed Student t test. n.s.=not significant.

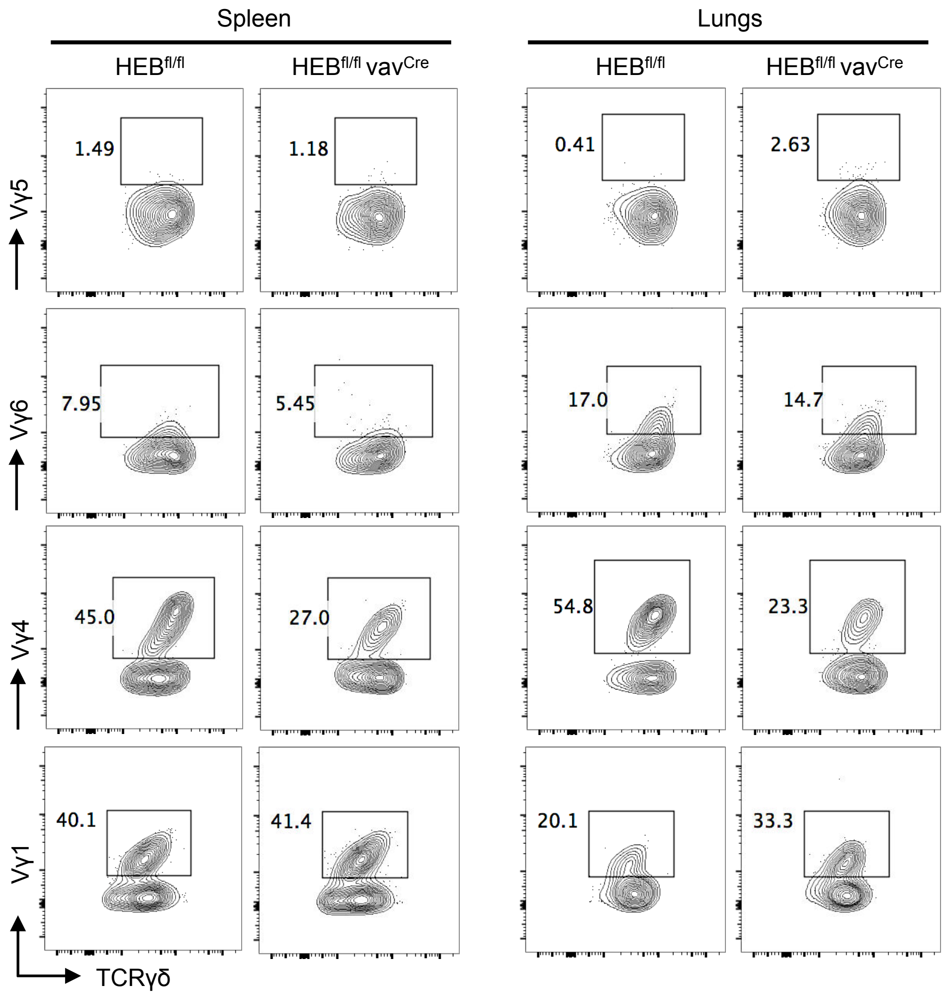

Supplementary Figure 9. Flow cytometric analysis of the Vγ repertoire in the periphery of HEB$^{fl/fl}$ vav^{Cre} mice. Vγ subsets in total splenic and lung γδ T-cells (CD3^{+}TCRγδ^{+}) as determined by analysis of surface Vγ expression. Vγ6 cells in these experiments were identified using the criteria of 17D1^{+}Vγ5^{-} staining within the γδ T-cell gate. 9 week old HEB$^{fl/fl}$ and HEB$^{fl/fl}$ vav^{Cre} mice were used for all analyses. Data are representative of at least three independent experiments with at least three mice per group.

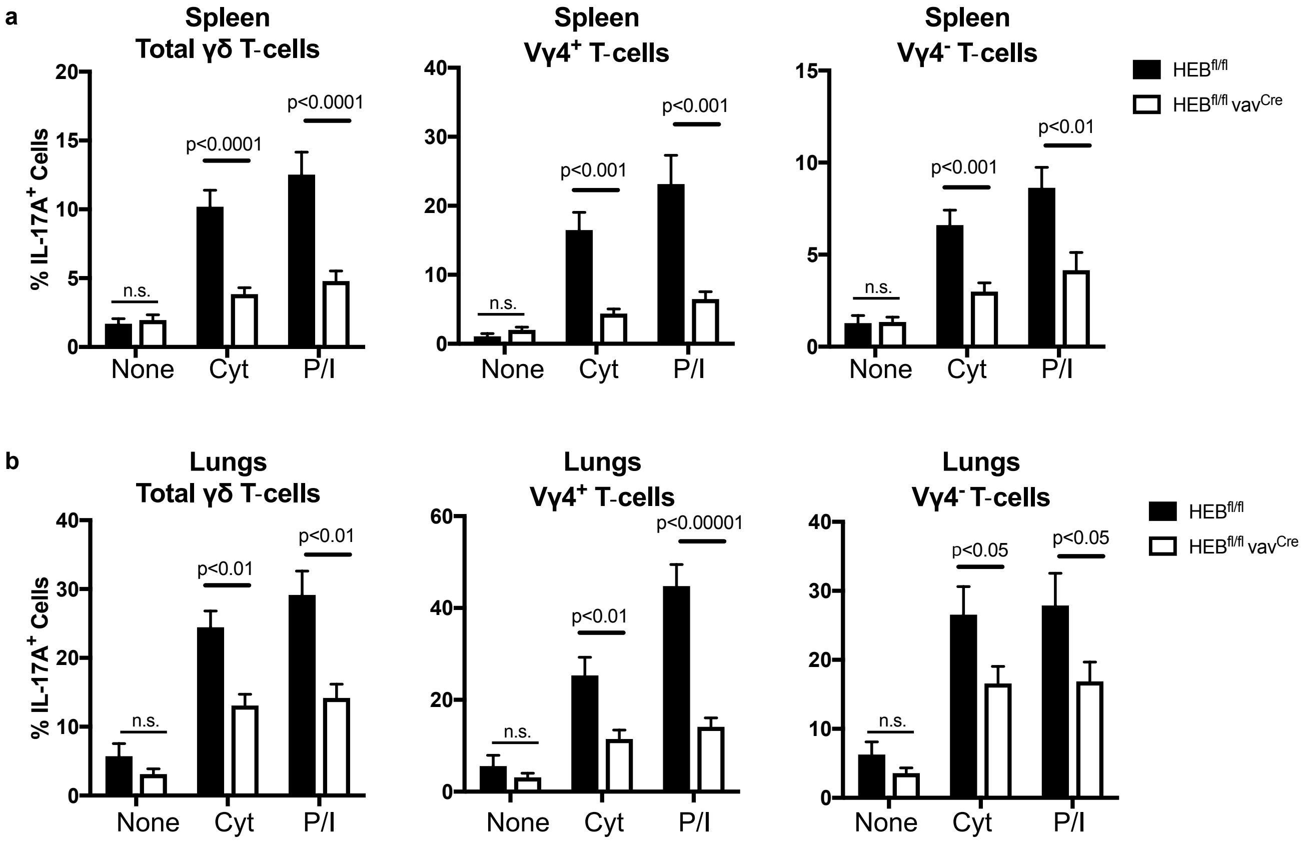

Supplementary Figure 10. Defective IL-17A production by Vγ4$^+$ and Vγ4$^-$ γδ T cells in the periphery of HEB$^{fl/fl}$ vav^{Cre} mice. a. Frequency of IL-17A$^+$ cells among total splenic γδ T-cells (CD3$^+$TCRγδ$^+$), Vγ4$^+$ cells (CD3$^+$TCRγδ$^+$Vγ4$^+$) and Vγ4$^-$ cells (CD3$^+$TCRγδ$^+$Vγ4$^-$), that had been stimulated for 5 hours with cytokines (IL-1β, IL-23, IL-21 and IL-7; Cyt) or PMA plus Ionomycin and IL-7 (P/I), as determined by intracellular staining. **b.** Frequency of IL-17A$^+$ cells in total lung γδ T-cells (CD3$^+$TCRγδ$^+$), Vγ4$^+$ cells (CD3$^+$TCRγδ$^+$Vγ4$^+$) and Vγ4$^-$ cells (CD3$^+$TCRγδ$^+$Vγ4$^-$) stimulated as in (a). Age matched 9-12 week old HEB$^{fl/fl}$ and HEB$^{fl/fl}$ vav^{Cre} mice were used for all analyses. Data are representative of at least three independent experiments with at least three mice per group. Values indicate mean, error bars denote mean+/-S.E.M. *p* values are given for all significant differences as determined by two tailed Student t test. n.s.=not significant.

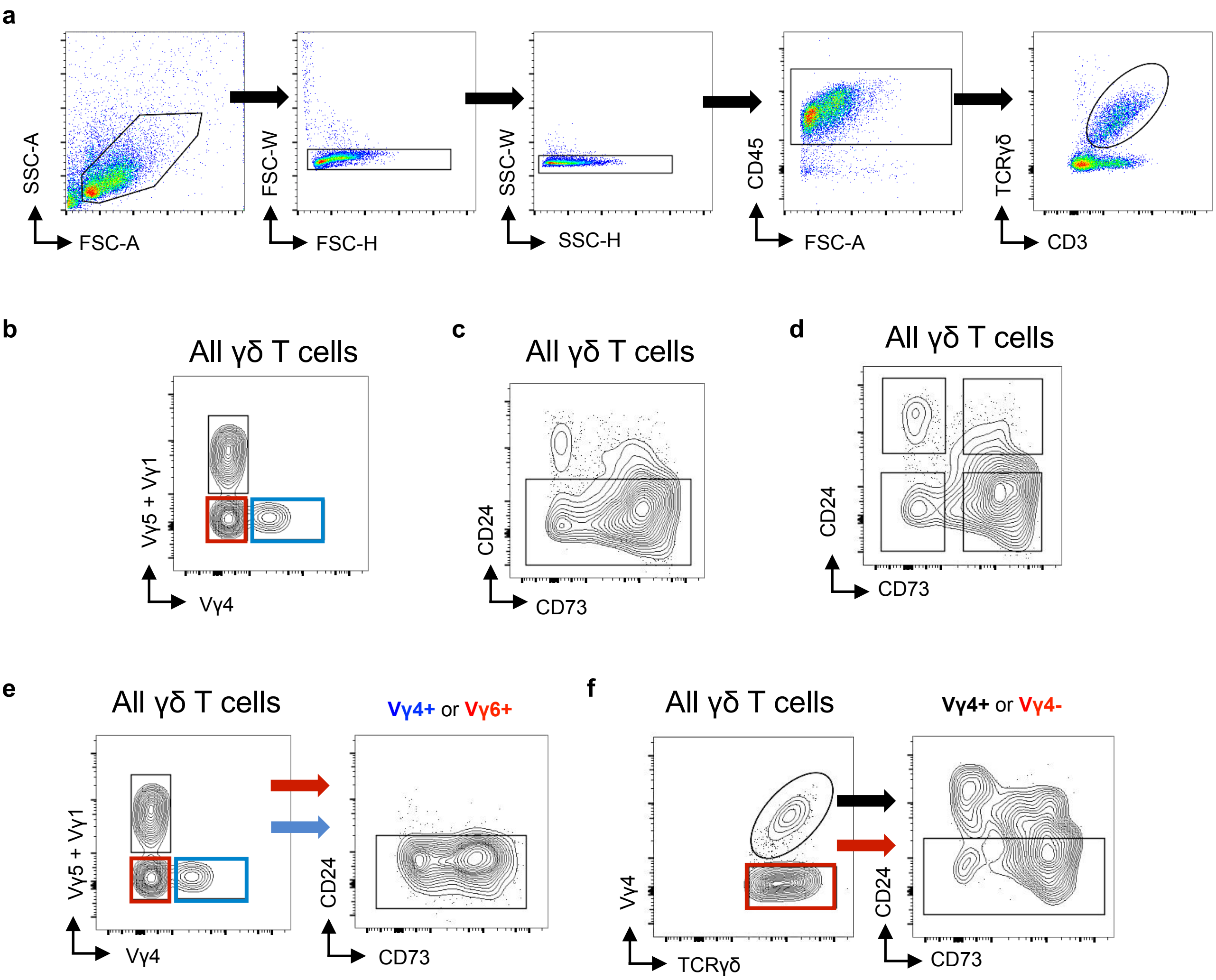

Supplementary Figure 11. Gating and sorting strategies for flow cytometry plots. a. Gating strategy to obtain "all γδ T-cells", as shown in Figures 1a, 1b, 1c, 1g, 2b, 3a, 4a, 7a, S1a, S2, S5a, S6a S7a, S7b, and S9. **b**. Gating strategy for Vγ1/5 ($V\gamma5^{+}V\gamma1^{+}$ shown in black), Vγ4 ($V\gamma4^{+}$ shown in blue), and Vγ6 ($V\gamma5^{-}V\gamma1^{-}V\gamma^{-}$ shown in red), within the "all γδ T-cells" gate, as shown in Figures 3a and 3c. **c.** Gating strategy for mature $CD24^{-}CD3^{+}TCR\gamma\delta^{+}$ cells within the "all γδ T-cells" gate as shown in Figures 4c and 4e. **d.** FACS sorting strategy for CD24/CD73 subsets within the "all γδ T-cell" gate analyzed by qRT-PCR in Figures 5 and S3. **e.** Gating strategy for Vγ4 ($V\gamma4^{+}$ shown in blue) $CD24^{-}$ cells and Vγ6 ($V\gamma5^{-}V\gamma1^{-}V\gamma^{-}$ shown in red) $CD24^{-}$ cells within the "all γδ T-cells" gate as shown in Figure 7e. **f.** Gating strategy for $V\gamma4^{+}$ (shown in black) $CD24^{-}$ cells and $V\gamma4^{-}$ (shown in red) $CD24^{-}$ cells within the "all γδ T-cells" gate as shown in Figure 8a. Flow cytometry plots of thymocytes from WT E14.5 FTOC day 7 and day 10 were used to demonstrate these gating strategies.

Supplementary Table 1. Primer sequences used for real-time PCR, luciferase reporter assay, and chromatin immunoprecipitation

Gene	Forward	Reverse
**qPCR primers**		
β-actin	ATGGTGGGAATGGGTCAGAA	TCTCCATGTCGTCCCAGTTG
Il17a	TCCAGAAGGCCCTCAGACTA	TGAGCTTCCCAGATCACAGA
Ifng	CTTCTTCAGCAACAGCAAGG	TGAGCTCATTGAATGCTTGG
Id3	CTGTCGGAACGTAGCCTGG	GTGGTTCATGTCGTCCAAGAG
Egr3	GACTCGGTAGCCCATTAGAATC	ACTTTCCCAAGTAGGTCACAG
HEBAlt	ATCCTGTCCCTGGAATGGGCAA	GATTCACGGTTGAAATCGTCAG
HEBCan	GGACTTCAGTGCGATGTTTTCT	ACGCTCATCCATACCTGACCCG
Sox4	GACAGCGACAAGATTCCGTTC	GTTGCCCGACTTCACCTTC
Sox13	GAACAGCAGCCACATCAAGA	TGCTGATGCTGGAGTTATGC
Rorc	ACAGCCACTGCATTCCCAGTTT	TCTCGGAAGGACTTGCAGACAT
Tbx21 (Tbet)	CCTCCTCTATCCAACCAGTATC	CTCCGGTTCATAACTGTGT
**Gibson primers for luciferase reporter assay**		
Sox13	CTGGCCTAACTGGCCGGTACCG CAGGTTCCCTTCCTCCTG	GAGGCCAGATCTTGATATCCTCG AGCAAGGCTGCTCAGAGGTTG
**Chromatin immunoprecipitation primers**		
GAPDH	TGGCGTAGCAATCTCCTTTT	CTCCTGGCTTCTGTCTTTGG
Sox4	ACACCTGGCACCGGAGACT	GAAGTGGCAGTTGGTGTGGA
